# Heterogeneity in radiation sensitivity within human primary tumour cell cultures as detected by the SCE assay.

**DOI:** 10.1038/bjc.1989.11

**Published:** 1989-01

**Authors:** P. J. Tofilon, C. M. Vines, R. E. Meyn, J. Wike, W. A. Brock

**Affiliations:** Department of Experimental Radiotherapy, University of Texas M.D. Anderson Hospital, Houston 77030.

## Abstract

The ability of the sister chromatid exchange (SCE) assay to detect heterogeneity in intrinsic radiation sensitivity was investigated. In order to identify tumour cell subpopulations, frequency histograms of cis-diamminedichloroplatinum (II) (cPt)-induced SCEs were generated and compared to those from cultures that had been irradiated 96 h before drug treatment. The results suggested that subpopulations with different radiosensitivities were present in nine of 18 human primary tumour cell cultures evaluated. When the effects of prior irradiation on the subsequent X-ray survival response and on cPt-induced SCE frequency histograms were compared, a good correlation was obtained between the two assays regarding the prediction of heterogeneity in radioresponse. These results suggest that primary cultures can contain both radiation-sensitive and radiation-resistant cells, and thus heterogeneity in intrinsic radiosensitivity may exist in human solid tumours.


*Br. J. Cancer* (1989), **59**, 54–60 © The Macmillan Press Ltd., 1989

### Heterogeneity in radiation sensitivity within human primary tumour cell cultures as detected by the SCE assay

P.J. Tofilon, C.M. Vines, R.E. Meyn, J. Wike & W.A. Brock

*Department of Experimental Radiotherapy, The University of Texas M.D. Anderson Hospital and Tumor Institute at Houston, 1515 Holcombe Boulevard, Houston, Texas 77030, USA.*

**Summary** The ability of the sister chromatid exchange (SCE) assay to detect heterogeneity in intrinsic radiation sensitivity was investigated. In order to identify tumour cell subpopulations, frequency histograms of *cis*-diamminedichloroplatinum (II) (cPt)-induced SCEs were generated and compared to those from cultures that had been irradiated 96 h before drug treatment. The results suggested that subpopulations with different radiosensitivities were present in nine of 18 human primary tumour cell cultures evaluated. When the effects of prior irradiation on the subsequent X-ray survival response and on cPt-induced SCE frequency histograms were compared, a good correlation was obtained between the two assays regarding the prediction of heterogeneity in radioresponse. These results suggest that primary cultures can contain both radiation-sensitive and radiation-resistant cells, and thus heterogeneity in intrinsic radiosensitivity may exist in human solid tumours.

The diversity of chemosensitivities among the cell subpopulations of a solid tumour is a well-accepted phenomenon, and its presence is considered to play a major role in a patient's ultimate therapeutic response. In contrast, the existence of heterogeneity with respect to intrinsic radiation sensitivity within a tumour not only remains in question, but the importance of such putative heterogeneity has been discounted. This is partially based on initial reports that mammalian cell lines have a relatively narrow range of $D_0$ values (the terminal slope of the radiation cell survival curve), leading to the assumption that the radiosensitivities of tumour cells do not vary sufficiently for the differences to have clinical significance (Berry, 1974). Consequently, intrinsic radiosensitivity has been largely ignored as a predictive factor in tumour radioresponse. Recent analysis indicates, however, that the critical survival curve parameter for predicting the *in vivo* radioresponse of human tumours is not the final slope ($1/D_0$) but the initial slope of the *in vitro* cell survival curve (Fertil & Malaise, 1981; Deacon *et al.*, 1984). Using the end-point of survival at 2 Gy as a measure of the initial slope, a broad range of radiosensitivities in both primary human cell cultures and established tumour cell lines has now been reported (Fertil & Malaise, 1985; Brock *et al.*, 1985a). Furthermore, the relative sensitivities of cultures derived from different tumour types generally correlate with the probability of clinical radiocurability as predicted by tumour histology (Fertil & Malaise, 1985; Brock *et al.*, 1985b). Thus, intrinsic radiosensitivity of tumour cells now appears to be a significant parameter in the clinical response to radiotherapy.

The mere presence of differences in *in vitro* radiation sensitivity among different tumours suggests that intrinsic radiosensitivity may also vary among the cell subpopulations of an individual tumour; such heterogeneity might be as clinically significant for radiotherapy as it is for response to chemotherapeutic agents. Recent attempts to develop methods for predicting human tumour radiosensitivity have concentrated on *in vitro* survival assays, yet radiation survival curves are not suitable for detecting minor resistant subpoplations of tumour cells. In fact, measurements of survival at the relatively low radiation dose of 2.0 Gy essentially reflect the response of the most sensitive cells in culture. Thus, to evaluate the diversity in cytotoxic agent sensitivity among cells of a single culture, it is necessary to use a method that assesses the response of individual cells. The sister chromatid exchange (SCE) assay, which is based on an analysis of individual metaphase cells (Wolff, 1981), meets this criterion. The induction of SCEs by specific antineoplastic drugs has been correlated with the induction of cell death: the SCE dose–response curves provide the same relative information as clonogenic cell survival measurements (Tofilon *et al.*, 1985, 1986; Deen *et al.*, 1986). In addition, we have shown that heterogeneity in drug sensitivity within a cell culture can be identified when SCE data are expressed as frequency histograms (number of cells versus SCEs/metaphase at a single drug dose), which illustrate the array of drug sensitivities among tumour cell subpopulations (Tofilon *et al.*, 1984a, b).

Because ionising radiation does not efficiently induce SCEs (Wolff, 1981), the SCE assay cannot be used to measure directly the radiation sensitivity of individual cells. Using a protocol that is based on the comparison of drug-induced SCE frequency histograms from previously irradiated and unirradiated cultures, however, it is possible to determine whether radiation kills cells in a non-random manner. In this protocol, SCE induction by an antineoplastic drug identifies the presence of tumour cell subpopulations; modification of the drug-induced histogram by prior irradiation then indicates that radiation preferentially killed a subset of the original population. In this case, in contrast to chemosensitivity, the SCE assay is used to provide an indirect measure of heterogeneity in radiosensitivity. To test the validity of his approach, we used a defined culture system composed of mixtures of two CHO cell lines with different radiosensitivities. These studies were then extended to human primary tumour cell cultures to determine whether intraneoplastic diversity in radiation sensitivity may potentially exist within human solid tumours.

### Materials and methods

### *Culture of established cell lines*

Two different strains of CHO cells were used: a wild-type line designated AA8 and its radiation-sensitive variant NM2 (van Ankeren *et al.*, 1989). Cultures of these cell lines were maintained in exponential growth as monolayer cultures in McCoy's 5A medium supplemented with 15% fetal bovine serum, 290 $\mu g\,ml^{-1}$ glutamine and antibiotics (100 units $ml^{-1}$ penicillin and 100 $\mu g\,ml^{-1}$ streptomycin). All incubations were carried out at 37°C in a humidified atmosphere of 95% air/5% $CO_2$.

### *Cell preparation and primary culture*

Biopsy and surgical specimens from human solid tumours were obtained from the Department of Pathology of The

Correspondence: P.J. Tofilon.
Received 23 May 1988; and in revised form, 11 August 1988.

University of Texas M. D. Anderson Hospital and Tumor Institute at Houston. Specimens were minced with scalpels and disaggregated into single cells by enzymatic procedures (Baker *et al.*, 1986). Cells were inoculated on to culture surfaces coated with a cell-adhesive matrix (CAM; LifeTrac, Irvine, CA). Growth medium consisted of Ham's F-12 with 4-(2-hydroxyethyl)-1-piperazine-ethanesulphonic acid, 10% swine serum and penicillin-streptomycin and supplemented with transferrin, hydrocortisone, epidermal growth factor and insulin as described. The initial attachment medium consisted of the above ingredients plus 0.6% methylcellulose. After 24 h of incubation at 37°C in a 91% air, 9% $CO_2$ atmosphere, the attachment medium was removed, the adherent cells washed with phosphate-buffered saline (PBS) and growth medium added.

*Treatment*

Irradiations for SCE analysis were performed at room temperature using a $^{137}$Cs source with a dose rate of 5 Gy min$^{-1}$. Primary cultures were irradiated when cells covered 10–20% of the culture surface (1–6 days of culture). CHO cells were irradiated 2 h after seeding. Stock solutions of cPt (*cis*-diamminedichloroplatinum (II)) dissolved in warm PBS and nitrogen mustard dissolved in 1 N HCl/ethanol were made immediately before use.

*SCE assay*

After drug treatment, cultures were rinsed with PBS, and 10 ml of fresh growth medium containing 10 μM bromodeoxyuridine (BrdU) was added. Culture dishes were wrapped in aluminum foil and the cells allowed to replicate for two cycles (28 h for CHO and 72 h for human primary tumour cell culures) before being harvested. Cultures were treated with Colcemid (0.04 μg ml$^{-1}$) 2 h before being harvested by trypsinisation (0.05% trypsin containing 1 mM EDTA). The cells were pelleted by centrifugation, resuspended in 0.075 M KCl for 15 min, then fixed and washed in freshly prepared methanol:acetic acid (3:1, v/v). Sister chromatids were differentially stained using the method of Perry & Wolff (1974). Fifty metaphase cells were evaluated in experiments using CHO cells and 20–50 metaphase cells were examined in experiments using human primary tumour cell cultures. The NM2 and AA8 CHO cell lines have the same number of chromosomes and thus data comparing these two cell types are expressed as SCEs/metaphase, whereas for human tumour cells, because of the wide variations in chromosome number among the cells of a primary culture, data are expressed as SCEs/chromosome. Because the number of metaphase cells that can be obtained from a single 100 mm$^2$ culture dish is limited and variable for primary cultures, each treatment group for primary cultures consisted of two or three culture dishes. Each dish was collected and analysed separately for SCEs. No significant differences were detected between the SCE values obtained from the individual culture dishes within a treatment group; the histograms shown in Figures 4 and 5 represent the pooled data from two to three culture dishes.

*Adherent tumour cell (ATC) radiation survival assay*

Designated 100 mm plates of human primary tumour cells were trypsinised (0.05% trypsin, 0.01 mm EDTA for 10 min), the cells were counted and the appropriate numbers seeded into CAM-coated 24-well multiwell plates for the performance of the radiation survival assay (Brock *et al.*, 1985b). Each set of cultures was irradiated with graded doses of 250 kVp X-rays (0–6.0 Gy). This was accomplished by irradiating a column of four wells at a time from the bottom of the dishes through a specially designed 3 mm lead collimator that allowed less than 3% scatter to adjacent culture wells. The cultures were then returned to the incubator, the medium was exchanged after 6 days and after a total of 13 days of incubation the cultures were washed with PBS and fixed with 70% ethanol. Ethanol-fixed cultures were stained by immersion of the entire multiwell plate in fresh 0.5% crystal violet for 10 min, after which time they were rinsed with water and allowed to air dry. The total staining density of each well was determined by integrating the absorbance over the entire surface of each culture well using a Magiscan 2 digital image analysis system (Joyce-Loebl, Gateshead, England). This measurement of staining density has been shown to reflect accurately the relative cell number in each set of cultures (Brock *et al.*, 1985b). Based on staining densities, surviving fractions were calculated as the fraction of treated wells relative to untreated control wells for each radiation dose, which allows for the construction of survival curves.

### Results

CHO cell lines with different radiosensitivities (AA8 and NM2) were grown independently or as mixtures in monolayer culture and then used to test the ability of combined radiation/drug SCE protocol to detect the presence of heterogeneity in radiation response. The mutant cell line, NM2, is more sensitive to cell killing by ionising radiation than the wild type AA8 cells: 10% survival for NM2 and AA8 results from 4.0 and 6.5 Gy, respectively (van Ankeren *et al.*, 1988). As reported for other types of mutant CHO cell lines (Thompson, 1985), NM2 is also more sensitive than wild-type cells to a variety of DNA-damaging drugs (van Ankeren *et al.*, 1988), including nitrogen mustard, an efficient SCE inducer. When NM2 and AA8 cells were treated with nitrogen mustard (0.05 μM for 0.5 h) and the SCE assay was performed, distinctly different SCE frequency histograms were obtained (Figure 1).

Although the SCE histograms for NM2 and AA8 cells overlapped, nitrogen mustard-induced SCEs still indicated the approximate percentages of each cell type within a mixed-cell culture, as shown by the top panels of Figure 2a and b, which are SCE histograms obtained from two different mixtures of the cell types. To determine the effect of prior irradiation on these heterogeneous populations, mixed-cell cultures containing AA8 and NM2 at ratios of 50:50 and 10:90, respectively, were seeded into monolayer culture and irradiated with 5 Gy 2 h later. Since this radia-

**Figure 1** Representative SCE frequency histograms for AA8 and NM2 cells after treatment with nitrogen mustard. Cultures were treated for 0.5 h with 0.05 μM nitrogen mustard, rinsed with PBS and fresh medium 10 μM in BrdU was added. Cells were harvested 28 h later and the SCE assay performed. For each histogram 50 metaphase cells were analysed.

tion dose results in approximately 5 and 20% survival for NM2 and AA8 cells, respectively, a preferential selection of AA8 cells would be expected. Cultures were then returned to the incubator for 48 h, which served as the 'selection period' (defined as the time allowed for both the expression of cell death and the proliferation of survivors). Unirradiated duplicate cultures were set up for each mixture. After the selection period, both irradiated and unirradiated cultures were treated with a relatively low dose of nitrogen mustard (0.05 $\mu$M, 0.5 h), and the SCE assay was performed. Representative nitrogen mustard-induced SCE frequency histograms generated for each mixture with or without previous irradiation are shown in Figure 2. In each case, prior irradiation with 5 Gy resulted in a shift to the left of the histogram, indicating an increased proportion of radioresistant AA8 cells, which thus reflects the selective killing of sensitive $NM_2$ cells and the continued proliferation of AA8 cells. These shifts were statistically significant as determined by the Mann–Whitney *U* test, which evaluates the differences between medians of two populations. These experiments were performed twice, with similar results obtained each time. Thus, this protocol identified the presence of heterogeneity in radiation sensitivity within a monolayer culture and was sufficiently sensitive to detect the presence of minor subpopulations (10%) of radiation-resistant cells (Figure 2b).

To investigate the possibility that human tumours contain cell subpopulations with different radiosensitivities, we applied a similar radiation/drug protocol to human primary tumour cell cultures. For human tumour cell cultures, rather than nitrogen mustard, cPt was used as the SCE-inducing agent to identify the presence of tumour cell subpopulations, as we have previously shown that primary human tumour cell cultures are frequently heterogeneous with respect to cPt sensitivity (Tofilon *et al.*, 1986). The specific radiation/cPt protocol is illustrated in Figure 3. On the day of biopsy, each primary culture was divided into four groups: group 1, control; group 2, 2.5 Gy only; group 3, cPt (5 $\mu$M) only; and group 4, 2.5 Gy followed 96 h later by cPt (5 $\mu$M). When cells covered approximately 10–20% of the culture surface, groups 2 and 4 received 2.5 Gy of $\gamma$-rays. The dose of 2.5 Gy was chosen because previous results using the adherent tumour cell survival assay showed that the most radiation-sensitive primary human tumour cell cultures have an approximately 10–20% survival level after 2.5 Gy (Brock *et al.*, 1985b). Thus, this dose of radiation should be sufficient to inactivate clonogenically the majority of any radiation-sensitive cells present. In these experiments, the 'selection period' (i.e. the time between irradiation and cPt) was 96 h, which corresponds to approximately two cell doublings. This length of time should be sufficient for surviving cells to proliferate and to increase their proportion within the culture, and to ensure that reproductively dead cells are no longer cycling and will thus not be included in the SCE analysis. After the selection period, groups 3 and 4 were treated for 1 h with 5 $\mu$M cPt to induce SCEs; all groups were then treated with BrdU and the SCE assay was performed. For primary cultures evaluated using this protocol, the histograms obtained after 2.5 Gy only (group 2) did not differ from their controls (group 1), indicating that, as expected (Wolff, 1981), radiation followed 96 h later by BrdU does not result in significant SCE inducation (data not shown).

Figure 4 shows the results from two different mesothelioma cultures that were treated and analysed according to this protocol. As the top panel of Figure 4a shows, specimen 0117 exhibited a fairly wide range in cellular sensitivities to cPt. Administration of radiation before drug treatment did not affect the distribution of cPt-induced SCEs in this culture (i.e. distributions in the top and bottom panels of Figure 4a are the same). For the other mesothelioma specimens (3936, Figure 4b), however, prior irradiation resulted in a large shift to the left of the cPt-induced SCE histogram, suggesting that radiation selectively reduced the number of

Figure 2 Representative SCE frequency histograms of cultures composed of mixtures of AA8 and NM2 after treatment with nitrogen mustard (0.05 $\mu$M for 0.5 h). Bottom panels in **a** and **b** represent cultures that received 5 Gy 48 h before treatment with nitrogen mustard. (**a**) AA8/NM2 mixture of 50:50. (**b**) AA8/NM2 mixture of 10:90.

Figure 3 Flow diagram of radiation/cPt protocol used on human primary tumour cell cultures.

**Figure 4** SCE frequency histograms obtained from two human primary tumour cell cultures after treatment with 5 μM cPt for 1 h. Bottom panels in **a** and **b** represent the group that received 2.5 Gy of radiation 96 h before cPt treatment. $n$ is the number of metaphase cells analysed. (**a**) Specimen 0117. (**b**) Specimen 3936. $P$ determined according to the Mann–Whitney $U$ test.

cPt-sensitive cells in 3936, which allowed the remaining cells (cPt-resistant) to emerge and dominate the cycling population of cells. This radiation-induced modification of the histogram is statistically significant. Thus, the results shown in Figure 4 suggest that culture 3936 was heterogeneous with respect to radiosensitivity and in culture 0117 heterogeneity was not present or, at least, not detectable.

The histograms obtained from six addtional primary cultures are shown in Figure 5. Again, the Mann–Whitney $U$ test, which essentially compares the medians of each group, was performed to determine whether the two histograms were significantly different ($P < 0.01$). The data in this figure show that radiation (2.5 Gy) did not significantly modify the cPt-induced SCE histograms for cultures 3942, 4010, and 3980 (Figure 5a–c). Prior irradiation induced a significant modification of the histograms obtained from cultures 4172, 3958, and 4046 (Figure 5d–f), suggesting that in these three cultures radiation selectively killed a subset of tumour cells. Thus, these data suggest the existence of heterogeneity in radiation sensitivity within four of the eight primary human tumour cell cultures depicted in Figures 4 and 5. It should be emphasised, however, that the lack of a radiation-induced modification of the cPt histograms shown in Figures 4a and 5a–c does not preclude the possibility that heterogeneity does exist in these cultures but was not detectable by our method (see Discussion).

If, as predicted by these SCE data, certain primary cultures are composed of cell subpopulations with different intrinsic radiosensitivities, then prior exposure to radiation should modify their subsequent survival response to X-rays. As a test of this hypothesis, primary cultures were treated with 2.5 Gy and 96 h later analysed for cPt-induced SCEs and their cell survival response to X-rays (Table I). Specifically, cultures were initiated and plates seeded for the SCE protocol as described in Figure 3, but including two additional plates. One of these plates received 2.5 Gy at the time of irradiation for the SCE protocol, while the other served as the untreated control. Ninety-six hours later, when the SCE plates were treated with cPt, these plates were trypsinised, the cells seeded into 24-well multiwell plates and the ATC radiation survival assay performed. Survival curves were then generated for the control and pre-irradiated plates and their intrinsic radiosensitivities expressed as survival at 2 Gy. The results are shown in Table I. Although the survival curves obtained from the ATC survival assay after irradiation of CHO cells are essentially the same as those obtained from the clonogenic assay (Brock *et al.*, 1985a), the precision of the ATC survival assay performed on human primary cultures has not yet been clearly defined. Thus, it was difficult to interpret the significance of small differences between the survivals at 2 Gy for the control and pre-irradiation groups for some of the cultures. We reasoned, however, that the same absolute change in survival between control and the pre-irradiated groups would be of greater significance in a sensitive culture (for example no. 0174) than in a resistant culture (e.g. no. 4186). Therefore, to account for the different radiosensitivities of the cultures evaluated, we expressed the change in survival at 2 Gy that results from prior irradiation as the per cent change (increase or decrease). Regarding the SCE data, rather than show the histograms for each culture, the effects of prior irradiation on the frequency of cPt-induced SCEs was expressed as the $Z$-value calculated from the Mann–Whitney $U$ test. The larger the $Z$-value, the greater the difference between the 2.5 Gy/96h/cPt and cPt only histograms.

When the influences of prior irradiation on survival measurements (per cent change in control survival at 2 Gy) and on cPt-induced SCE frequency histograms ($Z$-value) were compared, a correlation coefficient of 0.72 was obtained. Because we were concerned only with changes in survival and the SCE histograms, absolute values for per cent change in control survival and $Z$-values were used for comparison. To illustrate better the relationship between the change in cell survival at 2 Gy induced by prior irradiation and the heterogeneity in radiosensitivity as predicted by the SCE assay, the per cent changes in control survival levels were grouped according to whether or not a significant ($P < 0.01$) shift in the cPt-induced SCE frequency histogram was detected (Figure 6). Those cultures predicted to be heterogeneous with respect to radiosensitivity exhibited much larger changes in survival at 2 Gy as a result of prior irradiation. These data obtained from two different assays thus suggest, albeit indirectly, that primary cultures generated from human tumours can contain cell subpopulations with different intrinsic radiosensitivities.

### Discussion

Although the mechanisms of SCE formation have not been elucidated, SCE induction is considered to reflect DNA damage (Wolff, 1981). Since many antineoplastic drugs kill cells through direct damage to DNA, we hypothesised that SCE induction would correlate with cell killing (Deen *et al.*, 1986). To date, this has been shown for the chemotherapeutic agents cPt, BCNU and melphalan (Deen *et al.*, 1986; Tofilon *et al.*, 1985, 1986). A particular advantage of the SCE assay over currently available measures of cell killing is that it is based on the analysis of individual cells and can thus be used to evaluate the distribution of chemosensitivity among tumour cell subpopulations (i.e. heterogeneity). For example, in previous studies (Tofilon *et al.*, 1984*b*) various proportions of BCNU-sensitive and resistant 9L rat brain tumour cells were mixed in monolayer culture and treated

**Figure 5** SCE frequency histograms obtained for six human primary tumour cell cultures treated with 5 μM cPt (upper panel) and with 2.5 Gy 96 h before cPt (lower panel). Specimen numbers and tumour types are noted for each histogram. *n* is the number of metaphase cells scored. *P* was determined using the Mann–Whitney *U* test.

**Table I** Comparison of prior irradiation (2.5 Gy) on X-ray survival and cPt-induced SCEs

| | | *Survival at 2 Gy* | | | *cPt-induced SCEs* | |
|---|---|---|---|---|---|---|
| *Accession no.* | *Histology* | *Control* | *Pre-irradiated* | *% change*[a] | *Z-value*[b] | *n*[c] |
| 4186 | Ovarian carcinoma | 0.90 | 0.88 | −2.2 | 0.47 | 50 |
| 1054 | Melanoma | 0.82 | 0.84 | +2.4 | 2.06 | 21 |
| 4046 | Sarcoma | 0.59 | 0.37 | −37.3 | 4.13[d] | 30 |
| 1016 | Squamous cell carcinoma, head/neck | 0.48 | 0.45 | −6.3 | 0.68 | 50 |
| 1169 | Squamous cell carcinoma, head/neck | 0.42 | 0.43 | +2.4 | 0.69 | 30 |
| 1075 | Squamous cell carcinoma, head/neck | 0.38 | 0.35 | −7.9 | 0.05 | 21 |
| 4183 | Mammary carcinoma | 0.32 | 0.35 | +9.4 | 2.98[d] | 31 |
| 4172 | Adenocarcinoma, lung | 0.27 | 0.22 | −18.5 | 4.59[d] | 50 |
| 4188 | Squamous cell carcinoma, lung | 0.27 | 0.50 | +85.2 | 3.23[d] | 21 |
| 1036 | Squamous cell carcinomna, head/neck | 0.21 | 0.80 | +281.0 | 6.54[d] | 34 |
| 1074 | Melanoma | 0.13 | 0.18 | +38.5 | 4.06[d] | 31 |

[a]Calculated as (pre-irradiated − control/control) × 100.
[b]Determined from Mann–Whitney *U* test performed on SCE histograms.
[c]Number of metaphase cells analysed for cPt-induced SCEs in each histogram.
[d]$P < 0.01$ as determined by Mann–Whitney *U* test.

**Figure 6** Relationship between the heterogeneity in radioresponse present in primary cultures as predicted by the survival and SCE assays. Per cent change in survival at 2 Gy and the *P* values calculated from the SCE histograms are shown in Table I.

with BCNU. When the data were plotted as SCE frequency histograms, two regions corresponding to the BCNU-sensitive and resistant populations were obtained, and the approximate percentages of sensitive and resistant cells in each mixture could be predicted. Similar results were obtained from spheroids grown from mixtures of BCNU-sensitive and resistant cells (Tofilon *et al.*, 1984a). In addition, when primary cultures of human tumour cells were treated with cPt, some cultures that were sensitive, as predicted by their SCE dose–response curve and the $IC_{90}$ value from a survival assay, contained cPt-resistant cells as shown by SCE frequency histograms (Tofilon *et al.*, 1986).

In the present study, a culture system consisting of two different CHO cell lines, which simulated a human tumour culture composed of subpopulations with different radiosensitivities, was used to demonstrate that selective killing of a cell subpopulation by radiation could be detected using histograms of drug-induced SCEs. These studies using CHO cells as a model system supported the hypotheses that a shift of cPt-induced SCE frequency by prior irradiation indicates the coexistence of cell subpopulations with different intrinsic radiosensitivities within human primary tumour cell cultures.

The validity of this interpretation depends on the absence of any direct drug–radiation interactions affecting all or at least the majority of cells in the culture. Although a greater than additive level of cell killing has been reported for established tumour cell lines when cPt is administered just before irradiation in a combination protocol (Douple & Richmond, 1980), in our primary culture studies radiation was delivered 96 h before drug treatment, which corresponds to approximately two cell divisions. Because of this relatively long time interval between radiation and cPt treatment and because radiation preceded cPt, a direct interaction between these two agents is unlikely. Another possible complication with the interpretation of these data is due to a slightly greater sensitivity of $G_1$ cells to cPt's cytotoxic actions at more than 1 log cell kill (Meyn *et al.*, 1980) and to the radiation-induced growth inhibition at the $G_1$/S border (Okumura & Uchiyama, 1974). These factors could result in a cell-cycle synchronisation that could have been responsible for a shift in the SCE histograms. However, the cPt dose used to induce SCEs is very low with respect to cell killing and the radiation-induced growth delay, estimated at 0.5–2 h $Gy^{-1}$ (Okumura & Uchiyama, 1974), is not a significant factor in our studies, in which 96 h elapsed between radiation and drug treatment. Together these facts essentially eliminate the possibility that a radiation-induced cell synchronisation accounts for a change in the spectrum of cPt-induced SCEs. Therefore, changes in the drug-induced SCE histogram caused by prior irradiation appear to be the result of selective cell killing by ionising radiation.

The presence of tumour cell subpopulations with extremely different radiosensitivities should be detectable by a survival assay if the sensitive cells are first killed by an initial dose of radiation and then a selection period is given to allow surviving cells to emerge. This was clearly the case for three of the cultures shown in Table I (4046, 4188 and 1036), in which a large difference in survival was detected between the control and pre-irradiated groups. These results lend support to the prediction based on SCE data that these cultures were heterogeneous with respect to radiation sensitivity. In the other three heterogeneous cultures (4183, 4172 and 0174), as predicted by the SCE assay, only small changes in absolute survival were detected as a result of prior irradiation. These survival results expressed as absolute changes are more difficult to interpret and thus as supporting data are not as convincing.

In addition to absolute survival levels, however, we have also expressed the changes in survival in pre-irradiated samples as a percentage of the control value. This procedure, in essence, magnifies the effects of pre-irradiation in the radiosensitive cultures as compared to the more resistant cultures, which results in a better agreement between survival and SCE data regarding the prediction of heterogeneity (Figure 6). Whether this is an appropriate manner in which to express these survival data awaits future, more extensive work comparing the *in vitro* radiosensitivity as predicted by the ATC survival assay and clinical response. Indeed, the correlation between the heterogeneity predicted by the SCE assay and the change in survival for cultures 4183, 4172 and 0174 may be fortuitous. Alternatively, because the cell survival assay is dominated by the most radiosensitive cells within a culture, it is not expected to be as sensitive as SCE induction to the presence of heterogeneous subpopulations. Consequently, a relatively small change in survival may actually reflect the presence of subpopulations with very different radiosensitivities. Whatever the relationship between the survival and SCE measurements, it is clear that both assays indicate the existence of heterogeneity with respect to radiation sensitivity within some primary cultures. Although the SCE end-point may be a more sensitive indicator of heterogeneity, it should be emphasised that it does not predict the magnitude of the differences in radiosensitivity within a culture, only that differences exist.

Although modification of a histogram of cPt-induced SCEs suggests a heterogeneous radiation response, the absence of a modification does not eliminate the possibility that this form of heterogeneity existed in the original cell population. There are two possible explanations for such a situation. First, the sensitivity and accuracy of the SCE histograms in detecting minor subpopulations depends on the number of metaphase cells analysed. Since it was not possible to score more than 20 metaphases for some of the primary cultures, small subpopulations of tumour cells might not have been represented in the sample. We are currently modifying the SCE procedure in an attempt to obtain consistently a larger number of scorable metaphase cells from each primary culture. Second, detecting radioresponse heterogeneity with this SCE protocol is not possible when tumour cell subpopulations do *not* differ in their sensitivity to cPt. A range in cPt sensitivities is required in order to detect non-random killing by radiation within a primary culture. Thus, the heterogeneity detected by this technique is expected to be an underestimation. The question of intraneoplastic diversity in radiation sensitivity has, however, not been addressed to date, and despite its limitations, this SCE

protocol seems to provide information on this potentially significant biological parameter.

Based on the data shown in Figures 4 and 5 and Table I, a relationship between cPt and radiation sensitivity appears to exist in many primary cultures. Correlations between the radiation sensitivity of cells and their sensitivity to DNA-damaging drugs have been established using mutant CHO and murine leukaemia cell lines (Thompson, 1985; Sato *et al.*, 1986) and, specifically for cPt and radiation, using several human ovarian carcinoma cell lines (Louie *et al.*, 1985). With only one exception, in those primary cultures in which irradiation induced a shift in the cPt-induced SCE frequency histogram the shift was to the left, reflecting the emergence of cPt-resistant cells. In the exception, culture number 4046, irradiation resulted in a shift to the right of the cPt-induced SCE frequency histogram; however, pre-irradiation also resulted in a decrease in survival at 2 Gy, indicating an increase in radiosensitivity. While we cannot explain why prior irradiation resulted in an increase in the radiosensitivity of culture 4046, this result was predicted by the SCE assay and does support a positive correlation regarding the response of cells to cPt and radiation.

The range in radiation sensitivities for primary cultures and established tumour cell lines varies from 10 to 90% survival at 2 Gy (Deacon *et al.*, 1984; Fertil & Malaise, 1985; Brock *et al.*, 1985a, b). If this reflects the range of cell sensitivities within solid tumours, heterogeneity in intrinsic cellular radiosensitivity could be clinically significant. Because radiotherapeutic protocols usually involve the administration of thirty 2 Gy fractions, small variations in radiation sensitivity, if expressed in tumours, could be magnified into significant differences in tumour response. Based on the data presented herein using both the SCE and survival assays, it appears that heterogeneity in radiation sensitivity commonly exists in primary human tumour cell cultures and thus, perhaps, also in tumours. A clear understanding of the prognostic implications of intraneoplastic diversity in radioresponse as detected by the SCE assay awaits future clinical studies. However, it appears that the information concerning radiation heterogeneity obtained from this SCE protocol may complement radiosensitivity data from the ATC survival assay. Performing both of these assays on the same human primary tumour cell culture may ultimately form a basis for predicting tumour response to radiotherapy.

This work was supported by grant CA-06294 from the National Institutes of Health.

### References

BAKER, F., SPITZER, G., AJANI, J.A. & 8 others (1986). Drug and radiation sensitivity measurements of successful primary monolayer culturing of human tumor cells using cell-adhesive matrix and supplemented medium. *Cancer Res.*, **46**, 1263.

BERRY, R.J. (1974). Population distribution in tumors and normal tissues: A guide to tissue radiosensitivity. In *The Biological and Clinical Basis of Radiosensitivity*, Friedman M. & Thomas C.C. (eds) p. 141.

BROCK, W.A., MAOR, M.H. & PETERS, L.J. (1985a). Cellular radiosensitivity as a predictor of tumor radiocurability. *Radiat. Res.*, **104**, 290.

BROCK, W.A., WILLIAMS, M., BHADKAMKAR, V.A., SPITZER, G. & BAKER, F. (1985b). Radiosensitivity testing of primary cultures derived from human tumors. In *Proceedings of the Third International Meeting on Progress in Radio-Oncology*, Karcher, K.H. (ed), p. 185. Vienna.

DEACON, J., PECKHAM, M.J. & STEEL, G.G. (1984). The radioresponsiveness of human tumors and the initial slope of the cell survival curve. *Radiother. Oncol.*, **2**, 317.

DEEN, D.F., KENDALL, L.E., MARTON, L.J. & TOFILON, P.J. (1986). Prediction of human tumor cell chemosensitivity using the sister chromatid exchange assay. *Cancer Res.*, **45**, 1599.

DOUPLE, E.B. & RICHMOND, R.C. (1980). A review of interactions between platinum irradiation complexes and ionizing radiation: implications for cancer therapy. In *Cisplatin*, Prestayko, A.W., Looke, S.T. & Carter, S.R. (eds) p. 125. Academic Press: New York.

FERTIL, B. & MALAISE, E.B. (1981). Inherent cellular radiosensitivity as a basic concept for human radiotherapy. *Int. J. Radiat. Oncol. Biol. Phys.*, **7**, 621.

FERTIL, B. & MALAISE, E.B. (1985). Intrinsic radiosensitivity of human cell lines is correlated with radioresponsiveness of human tumors: Analysis of 101 published survival curves. *Int. J. Radiat. Oncol. Biol. Phys.*, **11**, 1696.

LOUIE, K.G., BEHRENS, B.C., KINSELLA, T.J. & 5 others (1985). Radiation survival parameters of antineoplastic drug-sensitive and resistant human ovarian cancer cell lines and their modification by buthionine sufoximine. *Cancer Res.*, **45**, 2110.

MEYN, R.E., MEISTRICH, M.L. & WHITE, R.A. (1980). Cell-dependent anticancer drug cytotoxicity in mammalian cells synchronized by centrifugal elutriation. *JNCI* **64**, 1215.

OKUMURA, Y. & UCHIYAMA, Y. (1974). A model of growth kinetics of irradiated cultured cells. *Int. J. Radiat. Biol.*, **26**, 321.

PERRY, P. & WOLFF, S. (1974). New Giemsa method for differential staining of sister chromatids. *Nature*, **251**, 156.

SATO, K., ITO, A., HIEDA-SHIORNI, N. SHIOMI, T. & HAMA-INABA, H. (1986). Cross-sensitivity to DNA-damaging agents in radiation-sensitive mutants of murine leukemia cells. *J. Radiat. Res.*, **27**, 378.

THOMPSON, L.H. (1985). DNA repair mutants. In *Molecular Cell Genetics: The Hamster Cell*, Gottesman, M.M. (ed). Wiley: New York.

TOFILON, P.J., BUCKLEY, N. & DEEN, D.F. (1984a). Detection of cell–cell interactions affecting growth rate and drug sensitivity in 9L multicellular spheroids. *Science*, **226**, 862.

TOFILON, P.J., WHEELER, K.T. & DEEN, D.F. (1984b). Detection of heterogeneity in chemosensitivity of 9L rat brain tumor cell lines to BCNU by the sister chromatid exchange assay. *Eur. J. Cancer Clin. Oncol.*, **20**, 927.

TOFILON, P.J., BASIC, I. & MILAS, L. (1985). Prediction of *in vivo* tumor response to chemotherapeutic agents by the *in vitro* sister chromatid exchange assay. *Cancer Res.*, **45**, 2025.

TOFILON, P.J., VINES, C.M., BAKER, F.L., DEEN, D.F. & BROCK, W.A. (1986). *cis*-Diamminedi-chloroplatinum (II)-induced sister chromatid exchange: An indicator of sensitivity and heterogeneity in primary human tumor cell cultures. *Cancer Res.*, **46**, 6156.

VAN ANKEREN, S., MURRAY, D., STAFFORD, P.M. & MEYN, R.E. (1988). Cell survival and recovery processes in Chinese hamster AA8 cells and 2 radiosensitive clones. *Radiat. Res.* **115**, 223.

WOLFF, S. (1981). Measurements of sister chromatid exchanges in mammalian cells. In *DNA Repair: A Laboratory Manual of Research Procedures*, Vol. 1, Part B, Friedberg, E.C. & Hanawalt, P.C. (eds) p. 547. Marcel Dekker: New York.


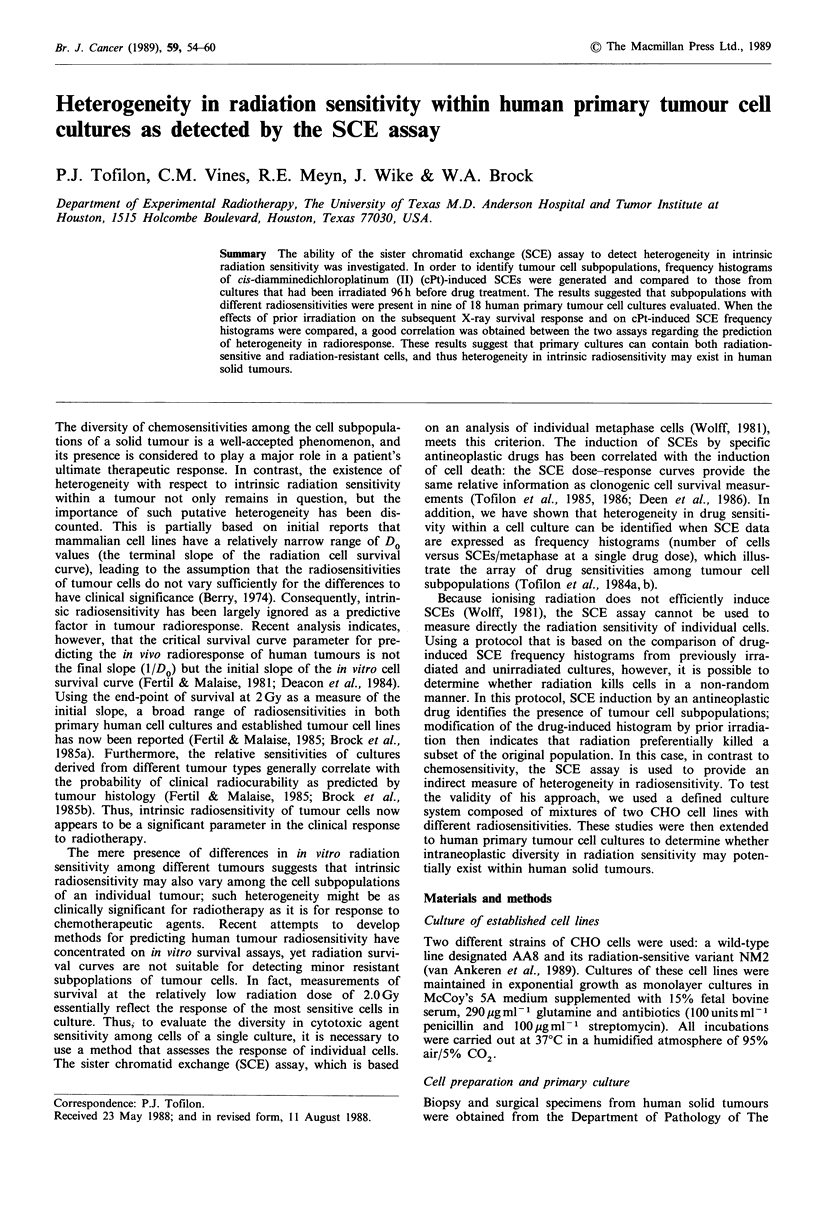

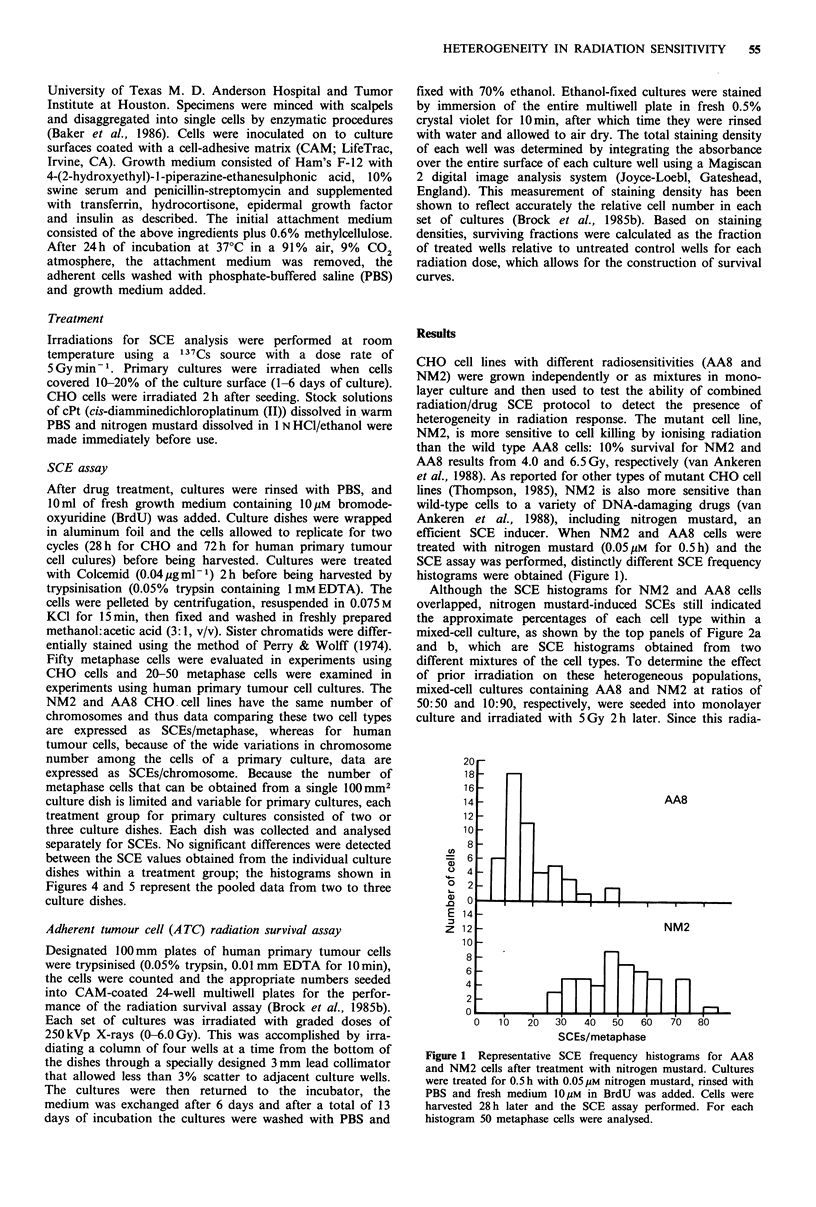

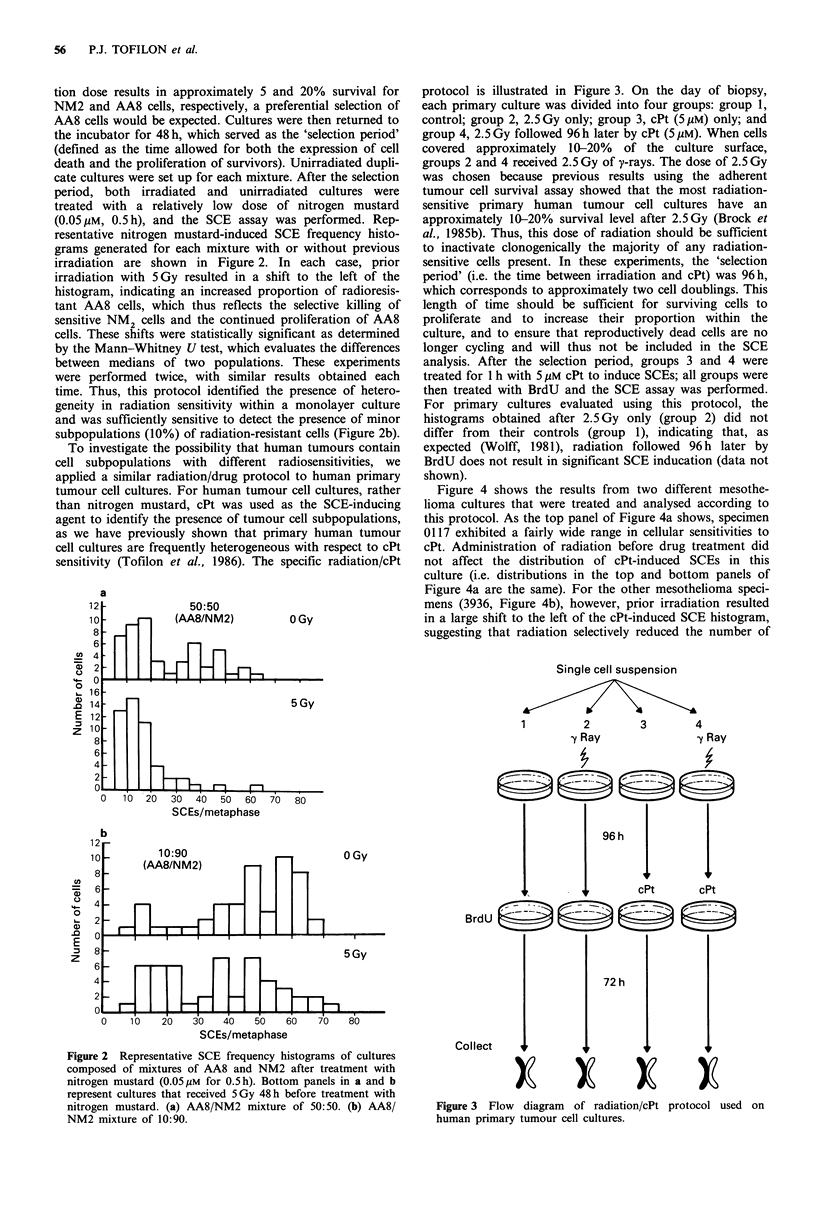

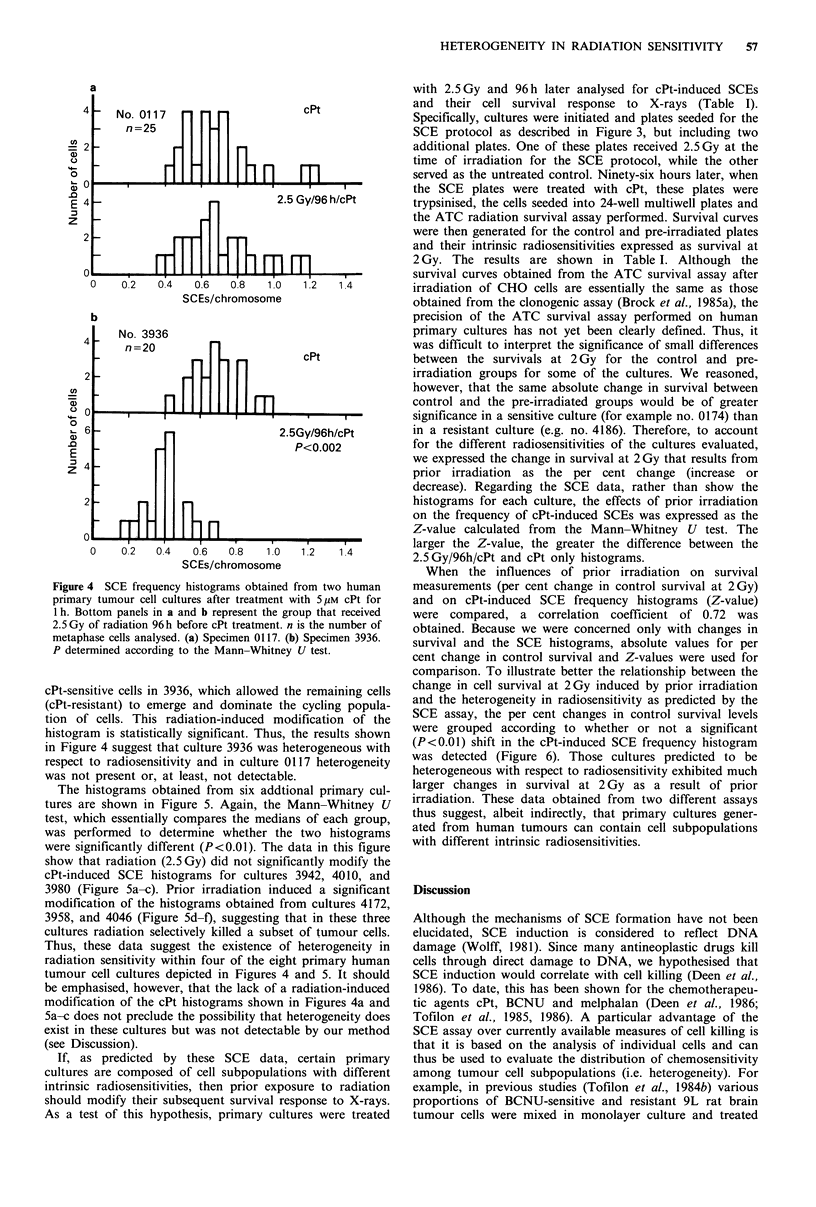

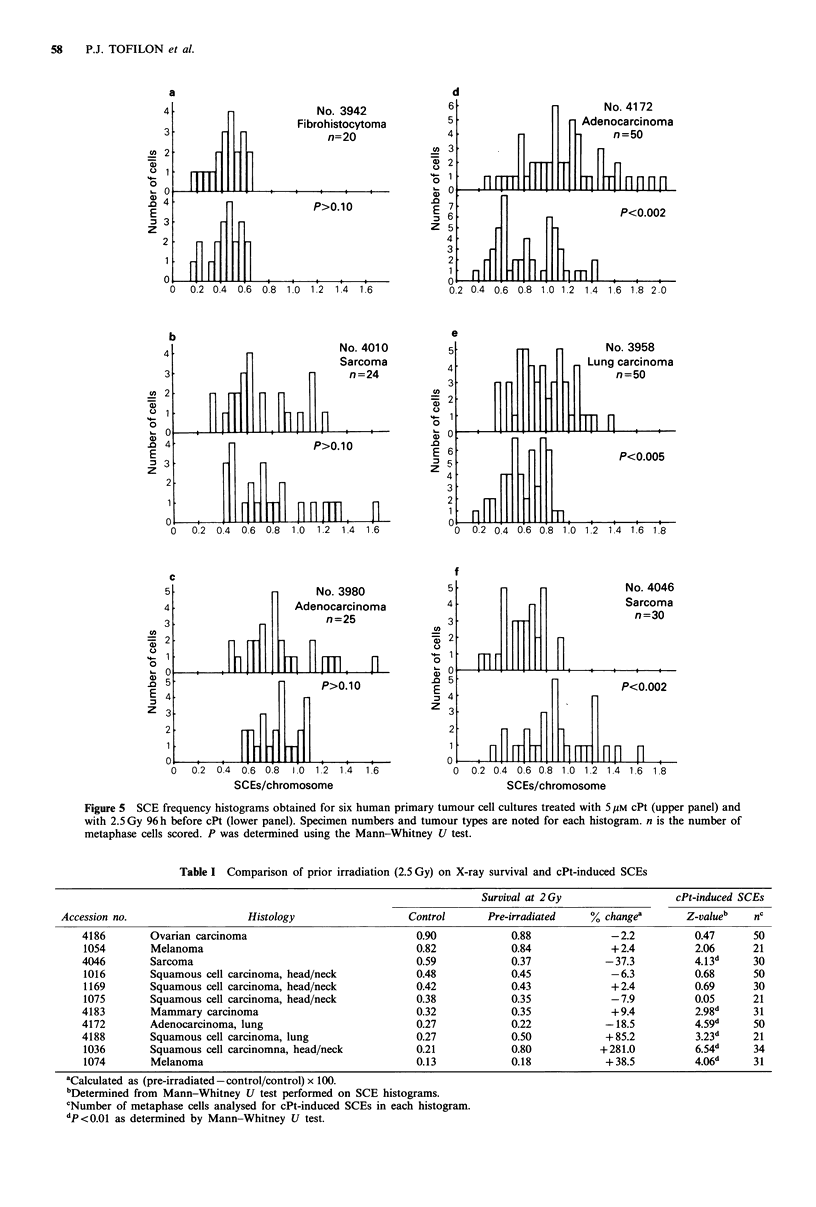

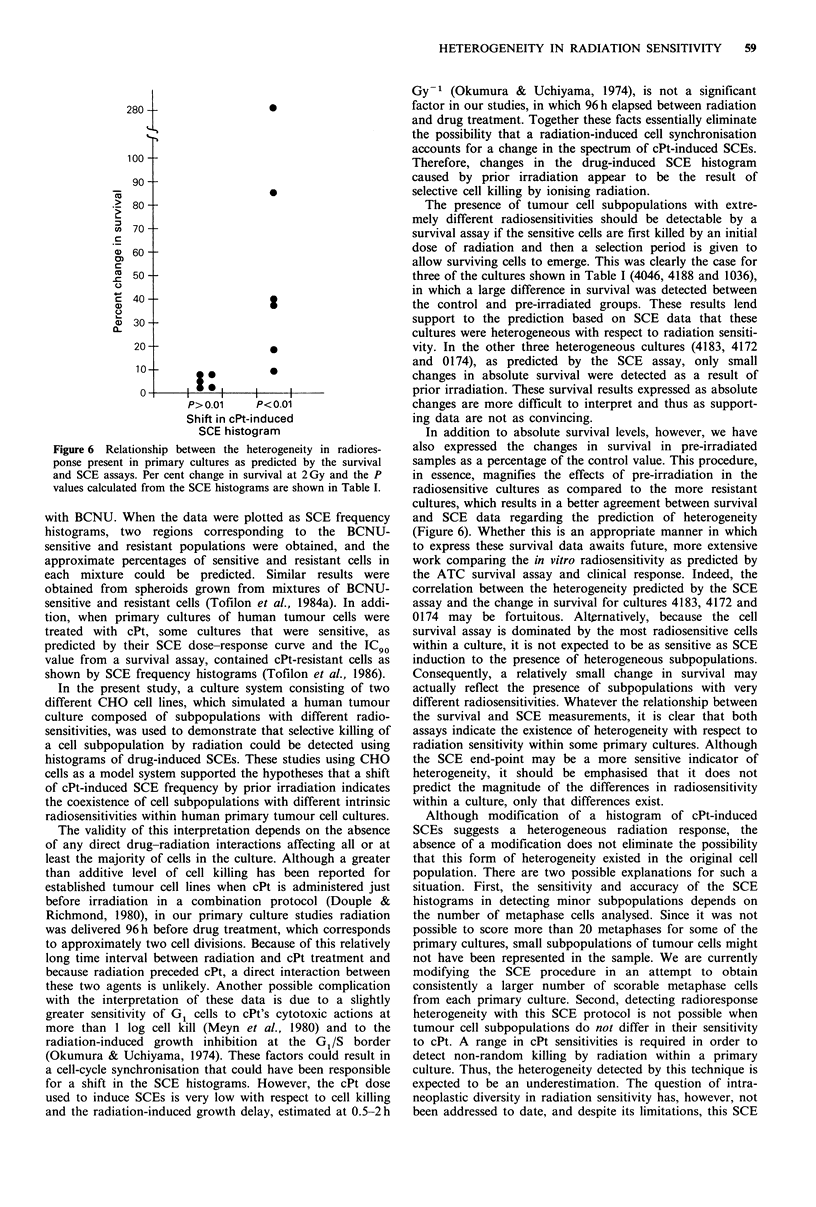

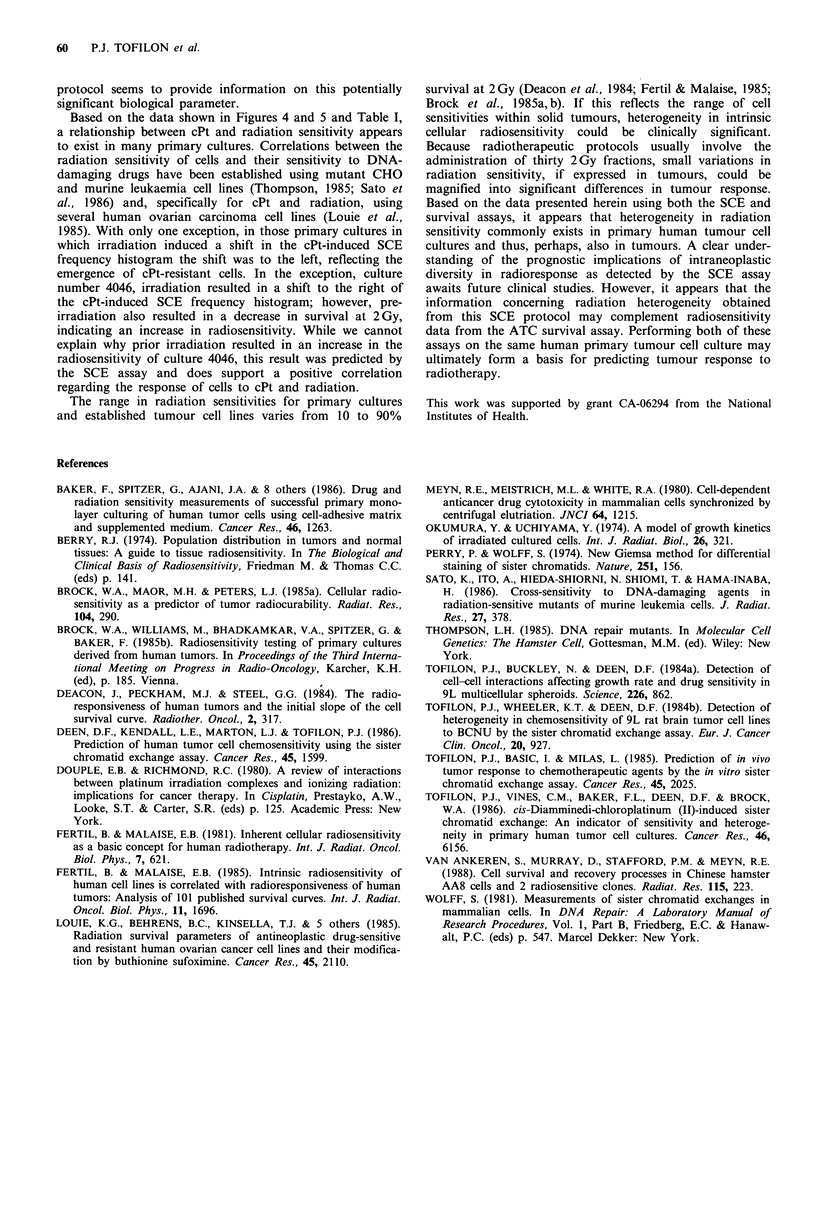


## References

[OCR_01032] Baker F. L., Spitzer G., Ajani J. A., Brock W. A., Lukeman J., Pathak S., Tomasovic B., Thielvoldt D., Williams M., Vines C. (1986). Drug and radiation sensitivity measurements of successful primary monolayer culturing of human tumor cells using cell-adhesive matrix and supplemented medium.. Cancer Res.

[OCR_01056] Deacon J., Peckham M. J., Steel G. G. (1984). The radioresponsiveness of human tumours and the initial slope of the cell survival curve.. Radiother Oncol.

[OCR_01061] Deen D. F., Kendall L. E., Marton L. J., Tofilon P. J. (1986). Prediction of human tumor cell chemosensitivity using the sister chromatid exchange assay.. Cancer Res.

[OCR_01073] Fertil B., Malaise E. P. (1981). Inherent cellular radiosensitivity as a basic concept for human tumor radiotherapy.. Int J Radiat Oncol Biol Phys.

[OCR_01084] Louie K. G., Behrens B. C., Kinsella T. J., Hamilton T. C., Grotzinger K. R., McKoy W. M., Winker M. A., Ozols R. F. (1985). Radiation survival parameters of antineoplastic drug-sensitive and -resistant human ovarian cancer cell lines and their modification by buthionine sulfoximine.. Cancer Res.

[OCR_01090] Meyn R. E., Meistrich M. L., White R. A. (1980). Cycle-dependent anticancer drug cytotoxicity in mammalian cells synchronized by centrifugal elutriation.. J Natl Cancer Inst.

[OCR_01095] Okumura Y., Uchiyama Y. (1974). A model of growth kinetics of irradiated cultured cells.. Int J Radiat Biol Relat Stud Phys Chem Med.

[OCR_01099] Perry P., Wolff S. (1974). New Giemsa method for the differential staining of sister chromatids.. Nature.

[OCR_01103] Sato K., Ito A., Hieda-Shiomi N., Shiomi T., Hama-Inaba H. (1986). Cross-sensitivity to DNA-damaging agents in radiation-sensitive mutants of murine leukemia cells.. J Radiat Res.

[OCR_01125] Tofilon P. J., Basic I., Milas L. (1985). Prediction of in vivo tumor response to chemotherapeutic agents by the in vitro sister chromatid exchange assay.. Cancer Res.

[OCR_01114] Tofilon P. J., Buckley N., Deen D. F. (1984). Effect of cell-cell interactions on drug sensitivity and growth of drug-sensitive and -resistant tumor cells in spheroids.. Science.

[OCR_01130] Tofilon P. J., Vines C. M., Baker F. L., Deen D. F., Brock W. A. (1986). cis-Diamminedichloroplatinum(II)-induced sister chromatid exchange: an indicator of sensitivity and heterogeneity in primary human tumor cell cultures.. Cancer Res.

[OCR_01119] Tofilon P. J., Wheeler K. T., Deen D. F. (1984). Detection of heterogeneity in the chemosensitivity of 9L brain tumor cell lines to 1,3-bis (2-chloroethyl)-1-nitrosourea by the sister chromatid exchange assay.. Eur J Cancer Clin Oncol.

[OCR_01137] vanAnkeren S. C., Murray D., Stafford P. M., Meyn R. E. (1988). Cell survival and recovery processes in Chinese hamster AA8 cells and in two radiosensitive clones.. Radiat Res.

